# How is maternal, newborn, and child health addressed in Heat Health Action Plans? A scoping review and content analysis

**DOI:** 10.7189/jogh.15.04157

**Published:** 2025-06-06

**Authors:** Alexandra Czerniewska, Chloe Brimicombe, Alejandro Saez Reale, Joy Shumake-Guillemot, Alyssa Sharkey, Anayda Portela

**Affiliations:** 1 Health Equity and Human Rights LLC, Princeton, USA; 2 Wegener Center for Climate and Global Change, University of Graz, Graz, Austria; 3 Global Heat Health Information Network Technical Support Unit, World Meteorological Organization, Geneva, Switzerland; 4 WHO-WMO Climate and Health Joint Office, World Meteorological Organization, Geneva, Switzerland; 5 Princeton University School of Public and International Affairs, Princeton, New Jersey, USA; 6 Department of Maternal, Newborn, Child and Adolescent Health and Ageing, World Health Organization, Geneva, Switzerland

## Abstract

**Background:**

Newborns, children, and pregnant and postpartum populations are among the most at risk from exposure to extreme heat. Heat Health Action Plans (HHAPs) are promoted by the World Health Organization to manage public health risks of heat. Still, limited research exists on how well current HHAPs address the needs of women and children in the context of increasingly frequent heat events.

**Methods:**

We identified national and subnational HHAPs published between January 2004 and July 2024 using various search channels. We extracted content to assess whether and how HHAPs included actions and indicators related to pregnant, postpartum, or breastfeeding individuals; newborns; and children.

**Results:**

We identified 83 eligible HHAPs from 24 countries, predominantly from high-income (49%) or lower-middle-income (47%) economies, with none from low-income economies. Most HHAPs identified children as a key population to protect (83%), with fewer naming pregnant individuals (52%). Even fewer mentioned newborns (39%) or postpartum and breastfeeding individuals (14%) as at-risk groups. We identified five broad activity categories targeting maternal, newborn, and child health (MNCH), with ‘informing, education, and awareness raising’ (77%) and ‘improving care in health services or school settings’ (59%) being the most common. However, no HHAP comprehensively addressed MNCH risks during extreme heat, and monitoring mechanisms were inadequate for assessing the impact of heat on MNCH.

**Conclusions:**

This is the first review mapping MNCH content in HHAPs. Comprehensive action plans must incorporate targeted strategies for at-risk MNCH populations to ensure equitable health outcomes during heat events. While many HHAPs focus on behaviour change messages, structural and policy changes are needed to build broader resilience. Strengthened in-country monitoring mechanisms and global support for better documentation are essential to build an evidence base.

Extreme heat conditions are having a growing impact on populations worldwide, increasing in occurrence, intensity, duration and area affected [1,2]. The World Health Organization (WHO) and the World Meteorological Organization (WMO) are among the organisations working to understand the at-risk populations and which interventions are most effective to reduce the health and social impacts of extreme heat. Extreme heat, including heat stress conditions (*i.e.* high humidity combined with raised temperatures) and heatwave periods of above average temperatures [3], increase morbidity in vulnerable groups and excess mortality, especially in persons over age 65 [4]. Compared to older people, the impacts of extreme heat exposure on maternal, newborn and child health (MNCH) have often been overlooked [5,6], but there is now a growing body of evidence that those who are pregnant, newborns, and children are also among the populations most at risk of adverse heat-related outcomes.

The association between maternal heat exposure and preterm birth, low birth weight, congenital conditions, stillbirth, and longer-term outcomes for the child has been established across many settings [7–12]. A 2020 literature review and meta-analysis [9] estimated a 5% increase in the odds of stillbirth and premature birth with each 1°C temperature increase and a 16% increase during heat waves, with the most significant associations in lower socioeconomic groups. Reviews also indicate that heat exposure during pregnancy, particularly during the second and third trimesters, is associated with physiological changes that heighten risk of pre-eclampsia, gestational diabetes, and other severe maternal outcomes [8], for example a 27% increase in the risk of serious illness (mortality ‘near misses’) during labour and delivery was observed among mothers with high exposure to heat during pregnancy in Southern California [13]. Infants and young children are uniquely affected by heat stress due to their developing physiologies, immune systems, and capabilities [8] associated with increased morbidity in the short-term [14,15], and long-term effects including reduced cognitive performance, lower adult earnings, and lower scores on academic tests [10,16]. However, we lack research from lower-income settings where exposures and risks are probably highest, and the evidence base for effective interventions to reduce risks to MNCH in extreme heat is also limited [17].

Heat health warning systems and heat health action plans (HHAPs) have existed for over 20 years, to develop and implement extreme heat adaptation and mitigation strategies tailored to the local context [18,19]. There has been a global mandate for their creation since the European heatwave in 2003 killed over 70 000 people [20]. They have had growing support through recent initiatives like the Global Heat Health Information Network, Anticipatory Action movement, UN Early Warning Systems for All [21], and the UN Call for Action on Heat [22]. The WHO Regional Office for Europe published the first comprehensive guide to the preparation of HHAPs by national, regional and local authorities in 2008 [23] – which was updated in 2011 [24] with an additional update expected in 2025 [25]. The guidance identifies eight core elements for comprehensive HHAPs: institutional coordinating mechanisms; heat–health warning systems; communication plans; care for vulnerable population groups; preparedness of the health and social care system; short- and long-term measures to reduce heat exposure; evaluation; and real-time surveillance of health outcomes. Initially, the guidance focussed on elderly populations and those with chronic conditions as vulnerable groups, and it was not until 2021 that the evidence on children and pregnant women as vulnerable groups was laid out in the context of HHAPs [25].

Previous research has assessed the coverage and aspects of the quality of HHAPs in parts of Europe [26,27] and other world regions [18,28]. There is no existing review of HHAPs, to our knowledge, which takes a MNCH perspective. Given increased awareness to consider the needs of MNCH during extreme heat conditions, the WHO commissioned this scoping review of national and subnational HHAPs to understand the extent to which plans currently aim to address and aim to monitor the effects of extreme heat on the health of those who are pregnant, postpartum, or breastfeeding, and on newborns and children. Our research questions were:

1. To what extent are MNCH populations considered at heightened risk from the health effects of extreme heat exposure in existing HHAPs?
2. What activities or actions in existing HHAPs specifically consider or target MNCH?
3. What indicators are currently in use to monitor the impacts of extreme heat on MNCH, or on general/other populations?

Therefore, our aim is to provide an overview of interventions included in current policy, to inform further work by the scientific, policy, and implementation communities in identifying what works to reduce the impacts on MNCH of extreme heat.

## METHODS

### Search strategy

We used several search channels to find HHAPs at national or subnational levels between 4 April and 31 July 2024. We issued a public call for plans through the Global Heat Health Information Network (GHHIN) email subscriber list. We sent email requests directly to health focal points in National Meteorological and Hydrological Services, WHO regional MNCH managers, members of a WHO working group on extreme heat and MNCH, and the HIGH Horizons project network. We searched publicly available databases of health-related planning documents such as GHHIN, Climate ADAPT, Reliefweb, and United Nations resources (*e.g.* National Adaptation Plans and Health and Climate Change Country Profiles). We reviewed reference lists and contacted authors of other recent reviews of HHAPs [18,26,28–31]. We also conducted Google searches using terms related to heat/heatwaves, climate, adaptation, plan/action, and the name of each country. Search terms were run in all local languages using Google Translate.

### Eligibility

For this review, we defined a HHAP as ‘a national or subnational, government-authored plan/strategy that considered the health risks of heat and presented a comprehensive set of prospective planned or recommended actions to reduce the effect of heat on human health, or to increase resilience to heat in the population’. Eligible HHAPs could be specific to heat and health, with titles such as ‘Heat Health Action Plan’ or ‘Heatwave Plan’, or multi-sectoral or multi-hazard plans if they contained a heat and health component, with titles such as ‘Adverse Weather Plan’, or ‘Health National Adaptation Plan’. Our definition took an intentionally flexible view of what a HHAP could be, since the development of heat/climate action plans is specific to different national and subnational contexts, but we included plans that attempted to include a breadth of considerations in keeping with the WHO concept of an HHAP [23,24] rather than documents describing a single programmatic plan (*e.g.* communication about health and health) or activity (*e.g.* a health-health warning system). We included HHAPs that covered all populations and sectors (*e.g.* a plan focussed only on managing heat stress in the workplace would be excluded). However, if links were provided to more detailed plans for MNC populations, we also reviewed these. We included the most recent version of plans identified, published since 2004 – the earliest examples of HHAPs following the 2003 European heatwave [18,32] – until 31 July 2024, in any language supported by Google Translate (**Table 1**).

**Table 1:** Eligibility criteria for inclusion

Criteria	Included	Excluded
Geography	National or subnational (*e.g.* regional, provincial, state, district, municipal from any country or territory)	Global or multi-country plans
Publication date	January 2004–July 2024 and the most recent version of plan	Plans before January 2004; previous versions of updated plans
Language	Any language supported by Google’s translation software	
Authorship	Endorsed/adopted by governmental body (*e.g.* national/local government ministry, public health institute); with or without another organisational author	Plans without a government author or co-author, judged by name or logo appearing on the document, or conversation with relevant authorities
Format	Any format (*e.g.* PDF report or webpage)	None
Type	Plans including a broad/comprehensive set of actions to protect human health in extreme heat; standalone heat health action plans, or in combination with other adverse weather events or climate change adaptation plans more broadly; masterplans or guidance documents (*e.g.* at national or regional level to guide subnational plan development)	Documents without planned or recommended actions for protecting human health in extreme heat; plans focussed on specific sectors, settings, or populations, or describing single programmes; retrospective reports, or presentations or articles describing a plan

### Data extraction and synthesis

Two reviewers (AC and CB) conducted data extraction and synthesis. AC led the data extraction, with CB independently reviewing a subsample of 10% of plans. Discrepancies were discussed and resolved with AP. Plans not originally in English were translated using the online translation software Google Translate. In rare cases where translations were unclear, or the precise translation was necessary (*e.g.* for defining the populations targeted), we used Google and Chat GPT to search for nuanced meanings of original words or sought clarifications from fluent speakers.

For each eligible plan, we extracted data on the geographical and administrative scope, the title and publication year, institutional authors, and language. We then reviewed each plan for MNCH content by reading relevant sections line by line (as determined by content tables) and searching whole documents for words related to MNCH such as ‘pregnan*’, ‘postpartum’, ‘postnatal’, ‘woman’, ‘women’, ‘breastfeed’, ‘lactat*’, ‘stillb*’, ‘f*etus’, ‘newborn’, ‘neonat*’, ‘birth’, ‘infant’, ‘baby’, and ‘child’, as well as terms related to our research questions more broadly such as ‘vulnerab*’, ‘risk’, ‘data’, ‘indicator’, ‘measure’, ‘monitor’, and ‘evaluat*’. We defined maternal health as during pregnancy, childbirth, and the postpartum period [33,34], including the health and development of the foetus up to birth, newborn health as the first 28 days after birth [35], and child health from one month to nine years old [36].

We used an iterative approach to data extraction, analysis, and mapping as per established scoping review methodologies [37]. A draft data mapping template was developed and tested against five plans and then finalised based on consensus between AC and AP. We started by coding content under three broad themes: MNCH risks, actions (*i.e.* interventions), and measures. We looked for whether and how these populations were considered to be at heightened risk from the health effects of heat exposure in the setting; any actions or activities recommended that specifically considered or targeted MNCH, and what, if anything, was measured or monitored for the general population and MNC populations. For example, monitoring heat exposures or vulnerabilities that exacerbate health effects, quantifying health, well-being, or social effects linked to the exposures, or tracking progress on implementing actions. To subdivide actions into different types, we initially considered a deductive approach using existing frameworks such as socio-ecological theories [38,39] to map how actions operate at different levels (*e.g.* individual, community, facility, policy levels), or by physical setting (*e.g.* home, health facility, school, and public space). However, we found that the actions were frequently described in insufficient detail to achieve this or led to overlap, and we therefore developed categories inductively based on the content identified, and then followed a process of inductively defining and refining data extraction fields based on themes we identified in the plans [40,41].

Our final mapping framework enabled us to look across four MNC population groups (*i.e.* newborns, children, pregnant women, and postpartum women), and count how many HHAPs categorised each population as high risk, included any of five defined types of interventions for each population, or included any of three defined types of monitoring measures for each population. These counts were assessed independently of each other. For example, if a HHAP defined both children and pregnant women as high-risk groups but only proposed a single type of intervention for pregnant women, then that HHAP would only contribute to the ‘counts’ in the corresponding parts of the framework.

## RESULTS

### Overview

We identified 83 plans from 24 countries (**Table 2**), representing 12% of the 195 WHO member countries, territories, and areas. We found that all 83 plans either named at least one MNC population as particularly at-risk from the effects of extreme heat (n = 70; 84%), included a planned activity targeted at least one MNC population group (n = 69; 83%), or did both (n = 57; 69%). Only 23 HHAPs (28%) included plans to monitor an indicator related to an MNC population.

**Table 2:** Description of eligible HHAPs, by WHO region, World Bank income group classification [42], administrative level, publication year, and original language (n = 83)

	HHAPs included, n (%)
**Countries by WHO region**	
African Region	1 (1)
South Africa (1N)	1 (1)
Americas Region	11 (13)
*Argentina (1S)*	1 (1)
*Canada (1R, 4S)*	5 (6)
*USA (3R, 2S)*	5 (6)
Eastern Mediterranean Region	2 (2)
Pakistan (1R, 1S)	2 (2)
European Region	26 (31)
*Austria (1N, 3R, 1S)*	5 (6)
*Belgium (1R)*	1 (1)
*France (1N)*	1 (1)
*Germany (1N)*	1 (1)
*Italy (1N)*	1 (1)
*Lithuania (1S)*	1 (1)
*Luxembourg (1N)*	1 (1)
*Netherlands (2N)*	2 (2)
*North Macedonia (1N)*	1 (1)
*Portugal (1N)*	1 (1)
*Spain (1N, 3R, 1S)*	5 (6)
*Sweden (1N, 2S)*	3 (4)
*Switzerland (1N, 1R)*	2 (2)
*UK (1N)*	1 (1)
South-East Asian Region	37 (45)
*Bangladesh (1N)*	1 (1)
*India (1N, 14R, 20S)*	35 (42)
*Nepal (1N)*	1 (1)
Western Pacific Region	6 (7)
*Australia (5R)*	5 (6)
*New Zealand (1N)*	1 (1)
**Country income level by World Bank classification**	
Low	0 (0)
Lower-middle	39 (47)
Upper-middle	3 (4)
High	41 (49)
**Administrative level**	
National	18 (22)
Regional	32 (39)
Subregional	33 (40)

HHAP – heat health action plan, N – national plan, R– regional plan, S – subregional plan, WHO – World Health Organization

We reviewed and excluded over 100 other documents. The most common reason for exclusion was a lack of an action plan for protecting human health in extreme heat (common among general climate adaptation plans), followed by documents that focussed on specific programmatic sectors, activities, or populations, such as clinical guidelines, frequently asked questions, or guidelines for certain industries only. We excluded one plan that we could not translate from Malayalam (Kerala, India, 2020).

We refer to all included plans as ‘HHAPs’ throughout, although 79 (95%) were specific to extreme heat, two (2%) encompassed heat and other adverse or extreme weather, and two (2%) were broader climate adaptation plans. Titles included ‘Heatwave Plan’, ‘Heat Action Plan’, ‘Excessive Heat Emergency Response Plan’, and ‘Guidelines on Heat-related Illness’.

**Figure 1:**
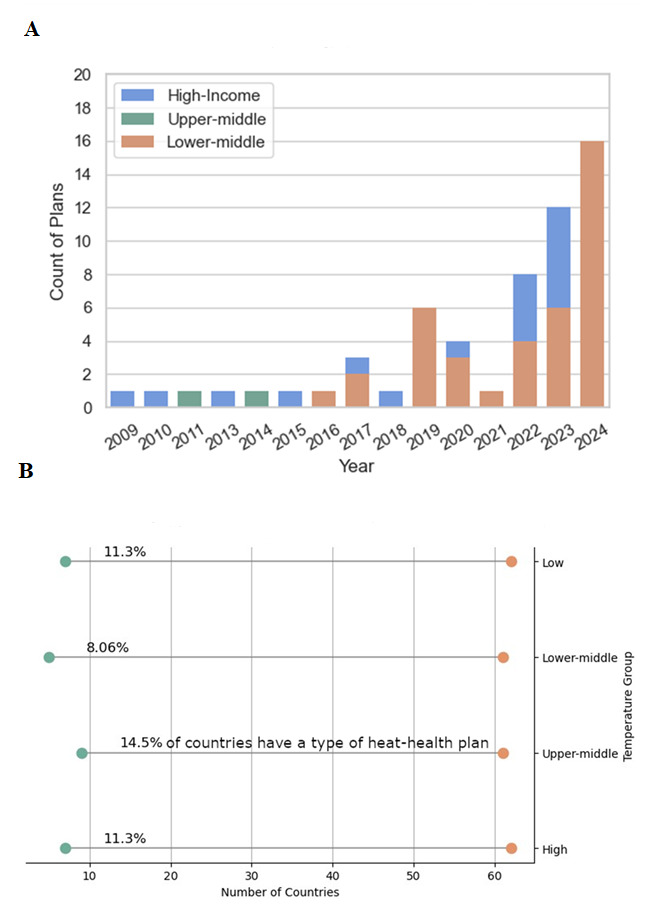
Distribution of heat-health plans by income group and temperature quantile.* **Panel A.** Income group. **Panel B**. Temperature quantile. *Temperature categories are constructed from climatology averages of annual average daily maximum temperature from 1991–2020 at a country-level aggregation [43] – low = <25th percentile, lower-middle = 25th–50th percentile, upper-middle = 50th–75th percentile, and high = >75th percentile.

The South-East Asian region contributed the most HHAPs overall (37 plans from three countries; 45%) including 35 HHAPs (42%) from Indian states, districts, and cities, but we identified HHAPs from the largest number of countries in the European Region (26 plans from 14 countries; 31%). The African region contributed 1 plan (1%), the Americas 11 plans (13%), the Eastern Mediterranean 2 plans (2%), and the Western Mediterranean regions 6 plans (7%). Almost all of the plans we identified were from countries classified as either high-income (n = 41; 49%) or lower-middle income economies (n = 39; 47%) – of which 35 were from India [42], and we found no plans from low-income countries. We found 18 national-level HHAPs (22%), 32 (39%) regional-level HHAPs – defined here as first-tier divisions below national-level, such as an Indian State or a Swiss Canton, and 33 (40%) at any levels below this, such as districts or cities (**Table 2**). HHAPs were published between 2009 and 2024, with 60 (72%) published in the past five years from 2020 (**Figure 1**, Panel A). The majority of HHAPs were originally in English (n = 59; 71%), however we included and translated plans in German (n = 6; 7%), Spanish (n = 6; 7%), French (n = 3; 4%), Swedish (n = 3; 4%), Dutch (n = 2; 2%), Flemish (n = 1; 1%), Italian (n = 1; 1%), Lithuanian (n = 1; 1%) and Portuguese (n = 1; 1%). We found that when assessing plans by temperature group constructed from the World Bank data set on climatological maximum temperatures aggregated to a country level, 11% of countries in the highest temperature category had some national or subnational HHAP (**Figure 1**, Panel B).

### MNC content – populations identified at risk

Across the 83 included HHAPs, 70 (84%) stated that at least one MNC population group was at increased risk from the effects of extreme heat: four (5%) HHAPs named all four MNC population groups; 25 (30%) HHAPs named three MNC populations; 24 (29%) HHAPs named two; 17 (20%) HHAPs named one, and 13 (16%) HHAPs named none (of these, 9 (11%) did not name any at-risk populations at all). Children were the most frequently mentioned MNC population group in 69 (83%) HHAPs (**Table 3**). Of these, 13 (16%) plans defined an age range for susceptible children, one stating children up to 14 years old, and the others up to four, five, or six years old. Newborns or infants were named as a population at increased risk in 32 (39%) plans. While our initial interest had been in the newborn population in the first 28 days of life, we ended up grouping terms such as ‘newborns’, ‘neonates’, ‘infants’, and ‘babies’ in this population group, since no plan gave a clear definition. People who are pregnant were identified as a high-risk group in 43 (52%) plans, with one plan specifying pregnant women over 35 or with co-morbidities as a particularly at-risk population subset (South Africa, 2020). People who were postpartum and/or breastfeeding were named in 12 (14%) plans. In 11 of these plans, the specific population mentioned was breastfeeding (or lactating) mothers. In contrast, one plan stated that mothers of young children were at increased risk due to increased socioeconomic vulnerability in this period (South Africa, 2020).

**Table 3:** Assessment of risk, activities, and monitoring, by population group (n = 83)*

	Newborns and infants	Children	Pregnant	Postpartum and/or breastfeeding	At least one MNC population
**MNC populations identified at risk**					
HHAP stated MNCH as a higher-risk population	32 (39)	69 (83)	43 (52)	12 (14)	70 (84)
**Categories of activities targeted to MNC populations**					
Informing, educating, and awareness raising	15 (18)	64 (77)	21 (25)	14 (17)	64 (77)
Providing direct material or financial assistance	0 (0)	15 (18)	3 (4)	1 (1)	15 (18)
Improving care in health services or school settings	11 (13)	47 (57)	12 (14)	5 (6)	49 (59)
Improving infrastructure in health services, community, or school settings	1 (1)	14 (17)	1 (1)	0 (0)	14 (17)
Improving working conditions for those who are pregnant/postpartum			16 (19)	0 (0)	16 (19)
**Measurement and monitoring for MNC populations**					
Outcome indicator	0 (0)	4 (5)	0 (0)	0 (0)	4 (5)
Process/other indicator	0 (0)	7 (8)	2 (2)	0 (0)	7 (8)
Other planned data collection	0 (0)	14 (17)	2 (2)	0 (0)	14 (17)

HHAP – heat health action plan, MNC – maternal, newborn, child *Presented as n (%).

### MNC content – activities

Most HHAPs (n = 69; 83%) included at least one activity specified for at least one MNC population group. However, there were 14 (17%) HHAPs which included no activities specified for MNC populations, despite all identifying one or more MNC population groups as high-risk. We grouped the activities identified in the plans into five categories; informing, educating, and awareness raising; providing direct material or financial assistance; improving care in health service or school settings; improving infrastructure in health service, community, or school settings; and improving working conditions for those who are pregnant/postpartum (**Table 3**).

The most identified activity category for all population groups was ‘informing, education and awareness raising’ (n = 64; 77%), followed by ‘improving care in health service or schools’ settings’ (n = 49; 59%) (**Table 3**). Activities for each of the other three categories were represented in approximately one fifth of plans: ‘providing direct material or financial assistance’ (n = 15; 18%); ‘improving infrastructure in health service, community, or school settings’ (n = 14; 17%), and ‘improving working conditions’ for people who are pregnant/postpartum (n = 16; 19%). Children were the most frequently targeted in each category (except improving working conditions, which was specific to people who are pregnant and postpartum), followed by people during pregnancy.

The most frequent MNCH activities under ‘informing, educating, and awareness raising’ were disseminating MNCH-specific messages via mass media and messages to children at school (File S1 in the **Online Supplementary Document**). Fewer HHAPs described MNCH messages communicated person-to-person by health workers or through community-level initiatives, such as encouraging neighbourhood or youth groups to check on the vulnerable MNC population (Western Sydney, Australia, 2018 and Bangladesh, 2024). Activities were most common in schools under the themes of ‘improving care’ and ‘improving infrastructure’, with fewer HHAPs including MNCH-specific activities for health or community settings. Four plans included interventions to improve care or infrastructure in maternity or newborn facilities during heatwaves, including three city-level plans from India (Surat City, 2023; Bhubaneswar City, 2020; and Rajkot City, 2022) with almost identical plans to relocate maternity or neonatal wards away from the hottest parts of buildings, increase education for parents in maternity wards before discharge, and revise the community health service training module on newborn care to include the management of heatwave conditions. Under ‘providing direct material or financial assistance’, one plan from a high-income setting mentioned a financial assistance scheme to support energy bills for air conditioning or evaporative cooling (Phoenix, Arizona, USA, 2024). The remaining were from the lower-middle-income settings of India and Bangladesh, and focussed on providing water, oral rehydration solution, and other low-cost items through the community health system or community groups. Under ‘improving working conditions for those who are pregnant/postpartum’, the plan from North Macedonia (2011) mentioned the possibility of policy-level interventions to protect pregnant workers. The plan from Styria, Austria (2023) recommended special consideration for pregnant women when evaluating work conditions in the heat and advised that occupational physicians should be consulted, and 14 plans from India included the wording on a ‘Dos and Don’ts for heatwaves’ list that 'pregnant workers and workers with medical condition should be given additional attention’ although no plans included detail on how to implement or monitor this.

No HHAP considered all four population groups across all five identified activity categories: 11 (13%) HHAPs (all from India) included an activity for at least one MNC population across four (n = 9; 11%) or five (n = 2; 2%) of the activity categories, while the modal number of categories covered was two (n = 26; 31%). Further, activities were often described with minimal detail, limiting efforts to understand how activities would be implemented or targeted to specific populations.

### Monitoring for MNC populations

Four (5%) HHAPs included plans to collect or monitor data for a health-related outcome with disaggregation by an age group, including or possibly including children: the regional plan from Vaud, Switzerland (2020) stated that data on number of visits to paediatric emergency departments would be stratified by under or over five years of age; the national plan from Spain (2023) stated that estimated deaths attributable to excess temperatures would be adjusted by age groups, with the youngest group being 0–14 years; the national plan for Portugal (2022) called for disaggregation of indicators including total number of primary health care consultations and total number of emergency hospital consultations by age group (groups not defined); the municipal plan for Madrid, Spain (2022) stated that all-cause mortality data would be disaggregated by age groups (groups not defined). An additional 14 (17%) HHAPs included tools indicating that age data would be collected, although they did not define an indicator or describe their planned analysis. For example, seven plans from India included standardised reporting forms for heat-related cases and deaths to be recorded at the facility level, including fields for age, sex, rural/urban location, and chronic conditions. These plans also included forms for district-level data aggregation by age (0–6 years and 7–18 years) and sex. No HHAPs included a health, well-being, or social outcome related to, or disaggregated by, newborns, pregnancy, postpartum period, or birth outcomes. Two (2%) HHAPs from Bangladesh (2024) and Karachi, Pakistan (2017) briefly mentioned the need to collect data on the pregnancy status of people affected by heat-related illnesses or death. The standardised health facility data-collection forms annexed to the Karachi plan included a space to record whether a patient suffering heat-related illness or death was pregnant.

Five (6%) HHAPs included a process indicator related to delivery of activities targeting children, *e.g.* number of child-friendly training sessions delivered in Vienna, Austria (2022) and Rajasthan, India (2017). Two (2%) HHAPs included a process indicator for those who are pregnant, *i.e.* the number who sign up to a ‘heatline’ alert system in Vienna, Austria (2022), and the number who become aware of heat-related illness in Rajasthan, India (2017). Two (2%) HHAPs stated they would monitor an indicator related to MNC heat vulnerability/ exposure, *i.e.* the number of children in poverty in England, (2024), and the proportion of the population under six years old in Thane City, India (2024).

To put this in context, across the 83 identified HHAPs, 47 (57%) stated that they would collect or monitor data for a health-related outcome for the whole population or a non-MNC population. No HHAPs defined an outcome indicator related to mental health or well-being, or included a social or economic outcome. Seven (8%) HHAPs included process indicators, and three (4%) HHAPs included monitoring of an exposure or risk indicator for the whole population (File S2 in the **Online Supplementary Document**).

### Sensitivity analysis

Due to the large proportion of HHAPs identified from India (43%), we conducted a sensitivity analysis comparing plans from India (n = 35) with plans from the rest of the world (n = 47) (File S2 in the **Online Supplementary Document**). The results indicated a comparable pattern of MNCH content across populations and risks, activities, and indicators, but with some differences in proportions (File S3 in the **Online Supplementary Document**). For example, Indian and non-Indian HHAPs were most likely to identify children as high risk and propose activities to protect children in the themes of ‘informing, educating, and awareness raising’ and ‘improving care in health care, community, or school settings’, however a higher proportion of HHAPs included these activities in the Indian plans: 97% *vs.* 63% and 80% *vs.* 40% for the two themes respectively. This was the case for almost all activity types and each population type. The HHAPs from outside of India were more likely to identify newborns and children as at higher risk (50% *vs.* 23% and 92% *vs*. 71%), but Indian plans more frequently identified pregnant and postpartum populations (60% *vs.* 46% and 23%* vs.* 8%). Evidence of monitoring and measurement was comparable between Indian and non-Indian plans.

## DISCUSSION

This scoping review explored the extent to which 83 HHAPs – including plans of any name which described actions for protecting human health in extreme heat – from 24 countries prioritised MNCH. Our purpose was to understand if and how the needs of these populations are currently considered in HHAPs, and to synthesise the extent and types of MNCH activities included. This review provides an overview of current policy at national and subnational level. Along with the findings from a parallel scoping review that mapped the literature on interventions implemented to reduce MNCH risk and heat [17], we hope to motivate the scientific, policy and implementation community to reflect on next steps, including how to monitor, document and evaluate the complex, multi-component and policy interventions needed, defining standard robust measures and evaluation methods to build the evidence base of what works for MNCH. We had initially planned to highlight examples from HHAPs providing potential ‘models’ of MNCH inclusion, including plans with diverse interventions across various MNC populations and activity types, and a clear intent to monitor the impact. However, no HHAP met this level of detail or consideration overall.

Our findings concur with previous work [28,38] that HHAPs and heat health research is unevenly distributed globally, and that populations identified to be most at risk of experiencing extreme temperatures and/or conditions exceeding human survivability [44,45] are not currently widely included in plans for monitoring or protecting human health in extreme heat. In alignment with Sustainable Development Goal 10 (*i.e.* reduced inequalities within and among countries), this suggests that tailored interventions that support low- and middle-income countries to develop and implement HHAPs are needed. Given the growing impact of extreme heat, we assume that more countries and subnational levels will develop HHAPs, and we therefore propose some recommendations for progressing MNCH inclusion in HHAPs

### The needs of pregnant and postpartum populations, newborns, and children should be identified in HHAPs

Most of the HHAPs identified children (83%) as a population at increased risk from extreme heat, and many also named those who are pregnant (52%). Fewer specified newborns/infants (39%) or postpartum or breastfeeding mothers (14%) as separate, at-risk populations. A growing recognition of the needs and heightened susceptibility of these groups in the context of heat and broader climate change reflects a growing body of effective global and local advocacy to highlight this [6,46]. However, our findings suggest that more can be done to ensure MNCH is systematically considered and prioritised in HHAPs.

Descriptions of at-risk child populations and associated activities were rarely framed with enough detail to distinguish needs across ages. There was a notable lack of actions across HHAPs to reduce risk to newborns. Only four plans included interventions to improve care or infrastructure in maternity or neonatal facilities or community care in the vulnerable newborn period. However, studies from high- and low-income settings have identified risks associated with exposure to extreme heat in the first days of life [47,48]. A mother’s exposure during pregnancy is associated with adverse birth outcomes, which may exacerbate risks to their newborns [9]. Many HHAPs referred generically to ‘infants’ without consideration of changing risks in the first weeks and months of life. Addressing the newborn in the home environment would be important. School-age children seemed to be the most frequent target of activities, mainly through school-based education and awareness raising. However, the literature suggests that the pattern of risk changes throughout childhood and adolescence and attention should also be given to pre-school children in HHAPs [8,49]. For example, a study from the USA examining the relationship between high temperatures and emergency department visits among children aged 0–4 found children aged 1–3 had the highest sensitivity to temperature increases, observing large surges in emergency department visits on the same day as temperature increases [50]. In contrast, for children under one year an increase in emergency department visits was observed on the third day after exposure. We join with other authors [6,49,51] to call for a more holistic and systematic consideration of newborn and child health in each stage of development in climate change planning and policy.

Few plans described efforts to improve pregnancy or birth outcomes – beyond communicating risks – such as targeted attempts to protect people in pregnancy who live or work in high-exposure conditions. Equally, there was no consideration of how to manage heat exposure during childbirth better, and the immediate postpartum period, considering the increased risks of infection with higher temperatures, especially in heat-prone regions. [52]. We found 12 plans that identified postpartum mothers as a high-risk group, with 11 of these specifying nursing/lactating mothers, although it was unclear in any of these plans whether the risk was considered for the mother, or, more likely, the effect on breastfeeding practices for the child [53] and no activities were planned to reduce either risk. Only one plan, from South Africa, referred to the risks specifically to women – linked to a higher likelihood of socioeconomic issues rather than any physiological risks.

#### HHAPs should integrate a broader range of relevant activities, specifically targeted to mediate the risks to MNCH

Across all HHAPs, we identified several planned activities targeted to different MNC populations across multiple settings and involving different governmental and non-governmental actors and delivery mechanisms; however, most individual plans included only a minimal number of activities or messages specific to MNCH, and many plans include none. This disconnect within HHAPs – between identifying several distinct vulnerable populations but then lacking formal actions and measures to protect them has been recognised by other authors [25,26] as an important focus for future efforts, and possibly suggests there is a window of opportunity for rapidly translating findings from ongoing and planned research into the most effective interventions for MNCH into policy.

Among the activities we identified, many put the onus on individuals to change their daily behaviours, primarily through information and messages distributed through mass media outreach and targeting schoolchildren with educational messaging as part of the school curriculum. Appropriate communication on heat and health risk to individuals and stakeholders is one of the main elements of the WHO guidance for HHAPs [24,25] and is crucial to ensure that at-risk people recognise their risk and take all possible preventative actions. However, WHO currently recommends that although the basic scientific content of messages is broadly correct, there is more work to be done to ensure messages are tailored and targeted to at-risk populations. They should be designed to change risk perceptions, capacity and opportunity to practice behaviours as well as increase knowledge, and delivered through a range of appropriate channels [25].

There were comparatively few structural or policy-level actions proposed for MNCH, such as preparing or strengthening health systems to respond to MNCH in extreme heat, creating multisectoral links with programmes such as in WASH or housing sectors, or state-level actions to protect pregnant, postpartum, or breastfeeding women in the workplace such as insurance/cash transfer schemes. Such a multisectoral focus would align with the United Nations’ Secretary General’s July 2024 Call to Action on Extreme Heat [22], which notes that policy responses are rarely multisectoral, as well as the United Nations’ Early Warnings for All Executive Action Plan [21]. Established global funding mechanisms, such as the Green Climate Fund, which prioritises financing climate adaptation and resilience efforts in vulnerable populations, and UN health programmes, particularly those under the Global Financing Facility, which focusses on improving health systems and advancing maternal, neonatal, and child health outcomes in low-income settings could help to bridge critical financial resource gaps for lower income countries.

HHAPs could consider how existing planned activities can be better accessed by, and tailored to, MNC populations and needs, *e.g.* creating specialised protocols for health services, guidelines for cooling centres that accommodate MNC needs, and communications campaigns with messages tested with different MNC populations. For example, many HHAPS described community cooling centres, but only one HHAP specifically discussed improving access to cooling centres for MNC populations. Measures such as separate spaces for breastfeeding, or facilities for children with toys and books available, could improve acceptability for MNCH populations [8], but only one plan mentioned that they would monitor which populations were using cooling centres. We note three examples where populations were highlighted: The Vienna Heat Action Plan (2022) specified the target populations for each proposed intervention, and the plans from Bangladesh (2024) and Surat City, India (2023) were framed explicitly in terms of consideration of gender considerations or MNCH populations.

#### The impact of heat on MNCH should be tracked with clear and standardised indicators and with specified periodicity

The monitoring mechanisms described within HHAPs were inadequate to support local and, much less, global understanding of the impact of a changing climate on MNCH and well-being. As written, these plans would not facilitate assessment of the effectiveness of interventions targeted at MNCH, nor would information be available to determine if the interventions were effective. We found very few plans (n = 5; 6%) describing how activities would be monitored regarding outputs or reach among MNC populations. We found no plans that described analysis of outcomes by whether a woman is pregnant or postnatal, or plans to monitor the effect of heat on pregnancy or birth outcomes. Four plans stated an intention to disaggregate morbidity or mortality data by age group. Still, in two of these plans, the age groups were not defined, and in the other two, the age brackets were too wide to monitor the variation in risk profiles over the different stages of childhood [54]. It is possible that such intentions are poorly described in HHAPs, and that health information systems are already collecting data that enable detailed and responsive analysis of the health impacts of heat across MNCH. However, for settings where new/parallel data reporting forms or data collection plans were described (*e.g.* in many of the Indian plans), it was often apparent that monitoring for MNCH groups would not be possible. UNICEF has recently recommended that indicators capture presenting cases of health-related illnesses by age group, gender and whether the individual is pregnant [8]. We propose that this also includes those who are postpartum and breastfeeding, and that age groups are narrow enough at young ages to capture data for newborns and in each year of early childhood.

We found that a handful of plans presented their selected indicators with definitions and data source, *e.g.* England (2024), Portugal (2022) and Spain (2023). Still, the lack of detail on measurement and monitoring across HHAPs meant we could not review how indicators for the general population would be defined or where data would come from. Many HHAPs (n = 47; 57%) referred to monitoring ‘heat-related’ illness/mortality within the general population. This was not defined but probably relates to directly-attributable conditions such as heatstroke, heat exhaustion, and heat rash/sunburn rather than including a range of physical conditions for which extreme heat could be a considerable contributing factor *e.g.* cardiovascular, renal, and respiratory admissions, water-borne disease, malnutrition, diarrhoeal disease [4,55,56], or mental health, well-being, or social outcomes [57,58]. Recent literature consistently finds surveillance, monitoring, and evaluation plans lacking in HHAPs [18,26,28] and broader climate change adaptation plans globally [29]. Martinez et al. [26] suggested that monitoring and evaluation in HHAPs had not improved in a decade, and we also noted that 17 of the 36 most recent plans – those published in 2023 and 2024 – included no planned outcome indicators. Guidance for the selection, definition, and use of indicators may be needed for the health impact of extreme heat, including guidance tailored to low- and middle-income country settings, where maintaining comprehensive, real-time surveillance may not be feasible. We note current efforts in developing global and national indicators for MNCH and extreme heat by the WHO and partners through the High Horizons Project and extensive work towards an update to the WHO guidance on HHAPs. Previous reviews have highlighted the range of climate-related health indicators in use globally and the need to better consider the usefulness and application of outcome indicators in each local context [59] and standardise heat exposure measures [60].

### Strengths and limitations

Two strengths of this review were our multi-channel strategy for identifying plans and our decision not to exclude plans based purely on scope or title. However, there was a limited number of publicly available plans. We could not analyse differences in MNCH representation in HHAPs across income levels, WHO regions or administrative levels due to the uneven distribution of identified HHAPs. Almost all plans were from high-income or lower-middle-income countries, and we found no plans from low-income countries. Further, most HHAPs included were from India, contributing 42% of all plans, 44% of all regional plans, and 61% of all subregional plans, and often contained near-identical wording around vulnerabilities, activities and monitoring. This pattern was also observed to a lesser extent in other countries. The dominance of HHAPs from India also skews our findings toward this region's approach and limits the generalisation of our findings. However, our sensitivity analysis demonstrated a similar pattern of content against our mapping framework within and outside of India and indicates that our key interpretations and messages would be the same with or without including the Indian plans.

We searched for HHAPS using several channels and included an extensive online search. Still, searching systematically by the names of all subnational areas (*e.g.* cities) worldwide was not feasible. Across the body of HHAPs we noticed that some mentioned activities at a high level and assigned a responsible agency/department to articulate and implement final plans, and others (usually at a national or regional level) provided a range of possible interventions for decentralised agencies to consider. In some HHAPs the challenges involved with defining governance and coordination processes [31] seemed to take precedence over laying out actions and monitoring plans, while other HHAPs included were public-facing documents, which provided detail about actions but perhaps less on the background workings of systems or surveillance. This may have led us to underestimate the support or monitoring planned for MNCH populations if activities were not well described and if MNCH populations were not explicitly mentioned. The results presented here demonstrate the extent to which protecting MNCH is explicitly planned for in HHAPs, but should not be read as an estimate of how effectively national and local governments protect MNCH during extreme heat.

## CONCLUSIONS

Integrating MNCH into HHAPs can enhance climate resilience in health systems and reduce heat-related risks for these populations, supporting Sustainable Development Goals 3 (*i.e.* health) and 13 (*i.e.* climate action). This review identifies substantial gaps in how HHAPs consider MNCH and calls for improved inclusion through targeted risk assessments, specific interventions, and robust monitoring. These findings also identify interventions included in HHAPs to inform global strategies, including the WHO’s Global Strategy on Health, Environment and Climate Change [61], the UN’s Global Strategy for Women’s, Children’s and Adolescents’ Health [62], and the WHO Europe’s Heat Health Action Plan guidance [24].

Existing HHAPs do not adequately recognise or address the specific needs of pregnant and postpartum individuals, newborns, and children during extreme heat. While children are often acknowledged as at-risk, detailed planning, resources, and tailored interventions are frequently lacking. Current plans focus on individual behaviour change over necessary structural reforms to build broader system resilience. Monitoring systems are inadequate for tracking the impacts of heat on MNCH or evaluating the effectiveness of interventions. While promising MNCH-related measures exist [5,7,8,52], they are applied inconsistently, and there is limited global guidance on effective interventions [17].

This review examines how HHAPs could better address MNCH needs, including integration into risk assessments, planning, and monitoring. At a minimum, local stakeholders should collaborate to test and adapt context-specific interventions and advocate for improved targeting of MNCH within existing HHAPs. Globally, the development of an operational framework is a critical next step. Such a framework should guide strategy, budgeting, and implementation, underpinned by integrated data systems to monitor impact, document costs, and share lessons learned. This will enable countries to better understand and address the vulnerabilities of MNCH populations to extreme heat, supporting effective and sustainable scale-up.

## Additional material


Online Supplementary Document
