# Supplementary material for: How is maternal, newborn, and child health addressed in Heat Health Action Plans? A scoping review and content analysis
Source: J Glob Health. 2025 Jun 6;15:04157. doi: 10.7189/jogh.15.04157 (PMC12143117; doi:10.7189/jogh.15.04157)
Supplement: Online Supplementary Document [file jogh-15-04157-s001.pdf]

Supplement to: Czerniewska A, Brimicombe C, Saez Reale A, Shumake-Guillemot J, Sharkey A, Portela A. How is maternal, newborn, and child health addressed in Heat Health Action Plans? A scoping review and content analysis. J Glob Health. 2025;15: 04157.

File S1. Monitoring for all populations

Across the 83 identified HHAPs, 47 (57%) stated that they would collect or monitor data for a health- related outcome for any/general population: 10 (12%) included a measure of heat-specific morbidity/mortality, and 18 (22%) included a measure of all-cause morbidity/mortality. Twelve plans [14%] included a combination of heat-related and all-cause outcomes. Of the HHAPs monitoring heat-related morbidity/mortality, XX plans from India specified cases and deaths from clinically-diagnosed heatstroke and all cardiovascular deaths, one plan from India (Ahmadabad, 2019) included monitoring water-borne diseases, malaria and dengue cases, and all other plans referred to general ‘heat-related’ morbidity/mortality without including a clinical or symptomatic definition. Where plans described types of data, these included primary health care visits, hospital visits and admissions (emergency department, intensive care units, or all hospital), death registers, calls to health helplines, and ambulance calls-outs. For example, the national plan from Portugal (2022) included monitoring primary health care and emergency department consultations, intensive care admissions, and deaths from all causes, alongside monitoring of heat-related calls to the national health service phone line where an algorithm could detect subjects related to heat, sunburn and sun exposure.

No HHAPs defined an outcome indicator related to mental health or wellbeing or included a social or economic outcome. Seven HHAPs (8%) included process indicators for general /non-MNC populations, for example related to delivery of messages and interventions, usage/understanding of interventions such as numbers of people accessing cooling centres, or spot checks on health facility stock. Three HHAPs (4%) included monitoring of an exposure or risk indicator including population density and slum population in Thane City, India (2024), percentage of population that could meet basic daily needs within a 400m walk/800m bike in Port Moody, Canada (2020), and the number of people living in ‘fuel poverty’ in England, UK (2004).

File S2.: Sensitivity analysis comparing (a) all plans, (b) plans excluding those from India,and (c) plans from India only. Conditional formatting has been applied to cells, with thedarkest shading indicating higher proportions of plans including the relevant risk, activity ormonitoring indicator.

(a) All plans (n=83)

	Newborns		Children		Pregnant women		Postpartum and/or breastfeeding		Any MNC Population	
**MNC populations at risk**										
Stated as a higher-risk population	32	(39%)	69	(83%)	43	(52%)	12	(14%)	70	(84%)
**Activities targeted to MNC populations**										
Theme 1: Informing, educating, and awareness raising	15	(18%)	64	(77%)	21	(25%)	14	(17%)	64	(77%)
Theme 2: Providing direct material or financial assistance	0	(0%)	15	(18%)	3	(4%)	1	(1%)	15	(18%)
Theme 3: Improving care in healthcare, community, or school settings	11	(13%)	47	(57%)	12	(14%)	5	(6%)	49	(59%)
Theme 4: Improving infrastructure in healthcare, community, or school settings	1	(1%)	14	(17%)	1	(1%)	0	(0%)	14	(17%)
Theme 5: Improving working conditions for pregnant/postpartum women					16	(19%)	0	(0%)	16	(19%)
**Evidence of measurement and monitoring for MNC populations**										
Outcome indicator	0	(0%)	4	(5%)	0	(0%)	0	(0%)	4	(5%)
Process/other indicator	0	(0%)	7	(8%)	2	(2%)	0	(0%)	7	(8%)
Other planned data collection	0	(0%)	14	(17%)	2	(2%)	0	(0%)	14	(17%)

(b) Excluding India (n=48)

	Newborns		Children		Pregnant women		Postpartum and/or breastfeeding		Any MNC Population	
**MNC populations at risk**										
Stated as a higher-risk population	24	(50%)	44	(92%)	22	(46%)	4	(8%)	44	(92%)
**Activities targeted to MNC populations**										
Theme 1: Informing, educating, and awareness raising	6	(13%)	30	(63%)	7	(15%)	2	(4%)	30	(63%)
Theme 2: Providing direct material or financial assistance	0	(0%)	2	(4%)	1	(2%)	0	(0%)	2	(4%)
Theme 3: Improving care in healthcare, community, or school settings	3	(6%)	19	(40%)	4	(8%)	0	(0%)	19	(40%)
Theme 4: Improving infrastructure in healthcare, community, or school settings	1	(2%)	4	(8%)	1	(2%)	0	(0%)	4	(8%)
Theme 5: Improving working conditions for pregnant/postpartum women					2	(4%)	0	(0%)	2	(4%)
**Evidence of measurement and monitoring for MNC populations**										
Outcome indicator	0	(0%)	4	(8%)	0	(0%)	0	(0%)	4	(8%)
Process/other indicator	0	(0%)	2	(4%)	1	(2%)	0	(0%)	2	(4%)
Other planned data collection	0	(0%)	7	(15%)	2	(4%)	0	(0%)	7	(15%)

(c) India only (n=35)

	Newborns		Children		Pregnant women		Postpartum and/or breastfeeding		Any MNC Population	
**MNC populations at risk**										
Stated as a higher-risk population	*8*	*(23%)*	*25*	*(71%)*	*21*	*(60%)*	*8*	*(23%)*	*26*	*(74%)*
**Activities targeted to MNC populations**										
Theme 1: Informing, educating, and awareness raising	*9*	*(26%)*	*34*	*(97%)*	*14*	*(40%)*	*12*	*(34%)*	*34*	*(97%)*
Theme 2: Providing direct material or financial assistance	*0*	*(0%)*	*13*	*(37%)*	*2*	*(6%)*	*1*	*(3%)*	*13*	*(37%)*
Theme 3: Improving care in healthcare, community, or school settings	*8*	*(23%)*	*28*	*(80%)*	*8*	*(23%)*	*5*	*(14%)*	*30*	*(86%)*
Theme 4: Improving infrastructure in healthcare, community, or school settings	*0*	*(0%)*	*10*	*(29%)*	*0*	*(0%)*	*0*	*(0%)*	*10*	*(29%)*
Theme 5: Improving working conditions for pregnant/postpartum women					*14*	*(40%)*	*0*	*(0%)*	*14*	*(40%)*
**Evidence of measurement and monitoring for MNC populations**										
Outcome indicator	*0*	*(0%)*	*0*	*(0%)*	*0*	*(0%)*	*0*	*(0%)*	*0*	*(0%)*
Process/other indicator	*0*	*(0%)*	*5*	*(14%)*	*1*	*(3%)*	*0*	*(0%)*	*5*	*(14%)*
Other planned data collection	*0*	*(0%)*	*7*	*(20%)*	*0*	*(0%)*	*0*	*(0%)*	*7*	*(20%)*
